# Lung cancer in the very elderly: incidence, presentation, and diagnostic decision-making. A retrospective analysis at a teaching community hospital

**DOI:** 10.3402/jchimp.v1i3.7313

**Published:** 2011-10-17

**Authors:** Sabarish Ayyappan, Claudia Gonzalez, Roopa Yarlagadda, Yousef Zakharia, Timothy J. Woodlock

**Affiliations:** 1 Department of Medicine, Unity Health System of Rochester, NY, USA; 2 University of Rochester School of Medicine and Dentistry, Rochester, NY, USA

**Keywords:** patient autonomy, medical decision-making, physician survey

## Abstract

**Background and objectives:**

Lung cancer presentation and decision-making in the very elderly patient population, 80 years of age and older, was studied given the projected increase in cancer incidence in the very elderly and yet only limited management guidelines.

**Design and setting:**

A 10-year experience at the Unity Health System of Rochester, NY, was reviewed using tumor registry data for the entire lung cancer population plus focused medical record review of very elderly patients. A questionnaire survey on the clinical approach to lung cancer in the elderly was distributed to medical staff involved in their care.

**Participants, measurements, and results:**

Of 997 patients, approximately 100 cases each year, the very elderly comprised 18% of patients from year 1998 through 2002, and 23% from year 2003 through 2007. One-third of the very elderly were diagnosed with lung cancer on clinical grounds without tissue confirmation. The majority of this group had cardio-pulmonary symptoms and an advanced clinical stage. The very elderly had no tissue sampling as per their own decision in 12 of 44 of cases, per family decision in 28 of 44, and per physician and other input in 4 of 44. Physicians stated that patient wishes and health-related factors, more so than socio-economic factors, were primary concerns for management decision-making.

**Conclusions:**

The number of very elderly lung cancer patients in this community setting has been significant and appears to be increasing. These patients were more likely to have an incomplete diagnostic work-up, with patient and family wishes being the major factor in medical decision-making. The physician approach to these patients emphasized patient autonomy and medical factors.

Lung cancer is the third most common cancer in the United States and the leading cause of cancer death both in women and men (1, 2). Tobacco smoke exposure, often the main cancer risk factor, precedes the onset of the clinical illness by years or decades and is cumulative (3). Hence, lung cancer is a concern in the very elderly, and many of our very elderly are seen in community hospitals for their lifetime care (4).

Our American population is aging (5). Accordingly, a marked increase in the number of cancer diagnoses in the elderly is expected over the next 20 years (6, 7). There were an estimated 222,000 new lung cancer patients in year 2010 and up to 338,000 new lung cancer diagnoses are estimated for year 2030, representing an approximate 50% increase in incidence. In 2030, it is expected that 271,000 lung cancer patients (80% of the total) will be over age 65 at time of diagnosis. This elderly lung cancer patient population is special because of their variable baseline health and comorbidities, limited involvement in clinical treatment trials to date, limited management guidelines, and potentially less enthusiasm for and higher risks with aggressive interventions. The American Society of Clinical Oncology supports efforts to identify the specific needs and management approaches for these elderly patients (8).

Given the above, we need a better understanding of our recent experience with elderly patients burdened with lung cancer, particularly the very elderly of 80 years of age and older, in the community hospital setting that cares for a large portion of the general population. Hence, we have studied patient-level data including demographics, diagnosis, symptoms, performance status, staging, and outcome of the lung cancer population at the Unity Health System of Rochester, NY over a 10-year span with focus on the very elderly patients. To investigate any trends in presentation and management, we have split the data set into two sequential 5-year increments: 1998 through 2002 and 2003 through 2007. Unity Health System provides comprehensive cancer care to a population of approximately 300,000 adults of mixed social and demographic background, encompassing city, suburban, and rural neighborhoods in upstate New York. The Unity Health System cancer program has been continuously certified by the Commission on Cancer of the American College of Surgeons throughout this time interval.

## Materials and methods

All patients diagnosed with lung cancer at Unity Health System from year 1998 through year 2007 were identified in the tumor registry. This registry is audited regularly for accuracy internally by the Unity Health System Cancer Committee and externally by the New York State Cancer Registry and the Commission on Cancer of the American College of Surgeons. As per guidelines of these organizations, cases are defined as lung cancer based on diagnostic clinical and radiologic findings as assessed by the attending physician without or with a confirmatory tissue sample (9). The very elderly in this study include patients 80 years of age and older. For comparison, diagnostic data from a separate regional community hospital and also from our university hospital were obtained from the Rochester Regional Tumor Registry.

In addition, with the approval of the Human Investigation Committee of Unity Health System, clinical records of selected patients were reviewed in a manner to protect patient confidentiality. Patient age and gender at diagnosis, diagnostic procedures, cancer histopathology, clinical symptoms, Eastern Cooperative Oncology Group (ECOG) performance status, clinical stage (*American Joint Committee on Cancer*, 6th edition), and survival were evaluated, along with comorbidities and selected laboratory data in the very elderly population. Data from the U S Social Security Administration were obtained to determine date of death when unavailable in the clinical records. It was not possible with this database to obtain a comprehensive cancer treatment history because some treatments were rendered outside of the institution with incomplete reporting to the Unity cancer registrar. However, initial treatment modality data were available and were reviewed for the very elderly patient population.

Data were tabulated in sequential 5-year increments, 1998 through 2002 and 2003 through 2007 to investigate any trends over time. The chi square test was used to compare patient populations. Survival was estimated by Kaplan-Meier analysis (10). A voluntary confidential survey questionnaire was distributed to our medical staff to review their approach to the very elderly with lung cancer.

## Results

A total of 997 patients were diagnosed with lung cancer at Unity Health System from year 1998 to 2007. As shown in Table 1, approximately one-half, 511 patients, were diagnosed from 1998 through 2002 and the remainder, 486 patients, from 2003 through 2007. There were near equal numbers of male and female patients in both time sets. The majority of patients were aged 70 years and greater, comprising 55% and 58% of the earlier and later time groups, respectively. The very elderly, 80 years of age and older, comprised 18% and 23% of the earlier and later groups, respectively, with a trend toward an increase over time (0.05 < *P*<0.01). There was a slight female predominance in the very elderly population, 57% (52 of 91) in the 1998–2002 group and 56% (62 of 111) in the 2003–2007 group (0.05 < *P*<0.1 for combined time periods). Of those with available documentation, 85% of the very elderly had a history of tobacco use. Initial diagnostic evaluation of lung cancer in the very elderly population occurred while the patient was hospitalized in over 90% of cases. Median survival was 4 months for the very elderly lung cancer patients diagnosed 1998 through 2002, and 2 months for those diagnosed 2003–2007.

**Table 1:** Lung cancer incidence by patient age and gender

Age	M	F	Total/%
*1998–2002*			
< 40 years	2	3	5/1
40–49	11	12	23/5
50–59	34	29	63/12
60–69	79	61	140/27
70–79	100	89	189/37
≥ 80	39	52	91/18
Total	265	246	511/100
			
*2003–2007*			
< 40	0	0	0/0
40–49	8	12	20/4
50–59	34	37	71/14
60–69	62	49	111/23
70–79	91	82	173/36
≥ 80	49	62	111/23
Total	244	242	486/100

Data are expressed as number of patients and were extracted from the Unity Health System tumor registry.

The proportions of non-small-cell and small-cell cancer for all patients with histopathologic diagnosis, 82 and 18%, closely match recent national data (2). In addition, a moderate proportion of patients, 17% of the total, were diagnosed by their physicians with lung cancer on clinical and radiologic grounds and reported to our cancer registrar as such without tissue biopsy data to confirm lung cancer. These patients were generally older. Four percent of the total patient population were less than 70 years of age and had no histologic diagnosis, whereas 13% were age 70 or greater and had no histologic diagnosis. Table 2 shows the age break-down of patients with histologic versus clinically defined lung cancer. For patients aged 80 years or greater, 26/91 = 29% had no histopathologic diagnosis for years 1998 through 2002, and 39/111 = 35% for years 2003 through 2007 (*P*=NS). For comparison, data from a separate regional community hospital and also from our university hospital were available through the Rochester Regional Tumor Registry for the years 2003–2007, showing 22 and 12% clinical diagnoses of lung cancer in the very elderly, respectively.

**Table 2:** Lung cancer diagnosis by patient age: histopathologic versus clinical definition

Age	Small cell *N*/%	Non-small–cell *N*/%	Clinical diagnosis *N*/%	Total *N*/%
*1998–2002*				
< 40 years	0/0	4/1	1/0	5/1
40–49	2/0	20/4	1/0	23/4
50–59	3/1	50/10	10/2	63/13
60–69	26/5	104/20	10/2	140/27
70–79	22/4	136/27	31/6	189/37
≥ 80	11/2	54/11	26/5	91/18
Total	64/12	368/73	79/15	511/100
				
*2003–2007*				
< 40 years	0/0	0/0	0/0	0/0
40–49	3/0	17/4	0/0	20/4
50–59	14/3	54/11	3/0	71/14
60–69	17/4	75/15	19/4	111/23
70–79	35/7	114/23	24/5	173/35
≥ 80	19/4	53/11	39/8	111/23
Total	88/18	313/64	85/17	486/100

Data were extracted from the Unity Health System tumor registry.

*N*=number of patients

**Fig. 1:**
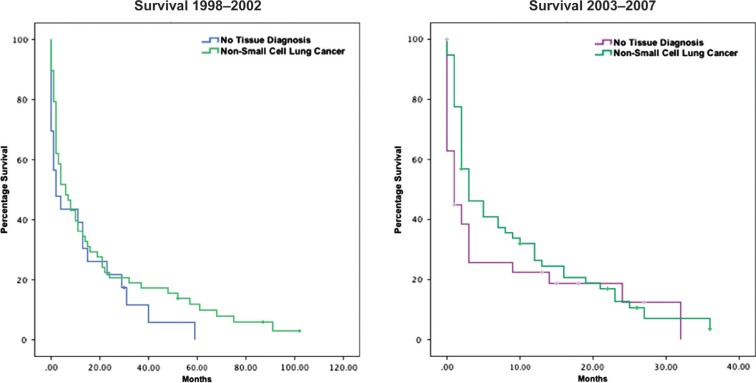
Survival of lung cancer patients aged 80 and over at Unity Health System by tissue histology, 1998–2002 and 2003–2007.

Survival was generally similar in the very elderly among those with non-small-cell and clinically defined disease (Fig. 1). Although there was a steep drop-off with a short median survival, approximately 20% of the patients with non-small-cell lung cancer and those with clinically diagnosed lung cancer lived 20 months or longer. Hence, a subset of each group had a favorable prognosis. Numbers of very elderly patients with small-cell lung cancer were too small to assess long-term survival. Also, patient records were not complete enough to confidently determine what clinical and laboratory factors were associated with longer survival. However, retrospectively, patients undergoing resection of the lung cancer were more likely to live over 12 months following diagnosis (10 of 11) versus patients without primary resection (23 of 65). No patient with a clinical diagnosis only of lung cancer received cancer treatment. Approximately 50% of the very elderly with a tissue diagnosis of lung cancer received some initial treatment – usually radiotherapy – with very few receiving initial cytotoxic chemotherapy.

Available hospital records of 49 of the 65 very elderly patients aged 80 years or greater with clinically defined lung cancer (no tissue) were reviewed. All of these patients were symptomatic on presentation; 20% had pneumonia syndrome as their chief complaint, 24% had dyspnea, 20% had cardiac symptoms, 8% were weak, 8% had skeletal events, and the remainder had CNS or abdominal complaints. Patient performance status was poor, ECOG 3 in 42% and ECOG 4 in 49% of patients. Four percent of these patients had clinical Stage II lung cancer, 23% Stage III, and 73% Stage IV (metastatic) disease versus approximately 50% local-regional and 50% metastatic disease in very elderly patients with a tissue diagnosis. Major comorbidities, including cardiopulmonary disease and diabetes, were present in all patients with clinically defined lung cancer where data were available. These comorbidities plus the acute presenting complaints compounded the patient's ill health and contributed to the poor global performance status. Pre-existing dementia was present in 20% of these patients. Significant anemia with hemoglobin concentration less than 11 g/dl was present in 29% of patients. From 1998 to 2002, 63% of clinically defined lung cancer patients were managed by primary care providers and 37% by hospitalists. From 2003 to 2007, 82% were managed by primary physicians and 18% by hospitalists. A minority of patients (6 to 9%) with a clinical diagnosis only of lung cancer had formal medical oncology evaluation.

Table 3 shows the responsible party for biopsy decision-making in the very elderly. Patients had no tissue diagnostic procedure by their own choice in 12 of 44 cases, by family decision in 27 cases, by physician decision in 3 cases, by health care proxy in one case, and by institutional ethics committee input in one case. Patients with dementia participated in decision-making in 17% of cases, whereas patients without dementia were involved in the diagnostic decisions in 45% of cases. For comparison, of the 137 very elderly patients with confirmed histopathologic diagnosis of lung cancer, records were available in 105 cases. Tissue was obtained in this population both for prognostication and treatment decisions and occurred while the patient was initially hospitalized in the vast majority of cases. These very elderly underwent tissue diagnostic procedures by their own decision in 39 of 105 cases, by patient and family input in 42 cases, by family in 23 cases, and by family and physician in one case.

**Table 3:** Decision-making for and against tissue diagnosis for clinically defined lung cancer in the very elderly

	No tissue N/%	Tissue obtained N/%
Patient	12/27	39/37
Patient and family	—	42/40
Family	27/61	23/22
Family and physician	—	1/1
Physician	3/7	—
Proxy	1/2	—
Ethics Committee	1/2	—

Data were obtained from available medical records of patients aged 80 years or greater presenting to Unity Health System with clinical and radiologic evidence of lung cancer.

N=number of patients

**Fig. 2:**
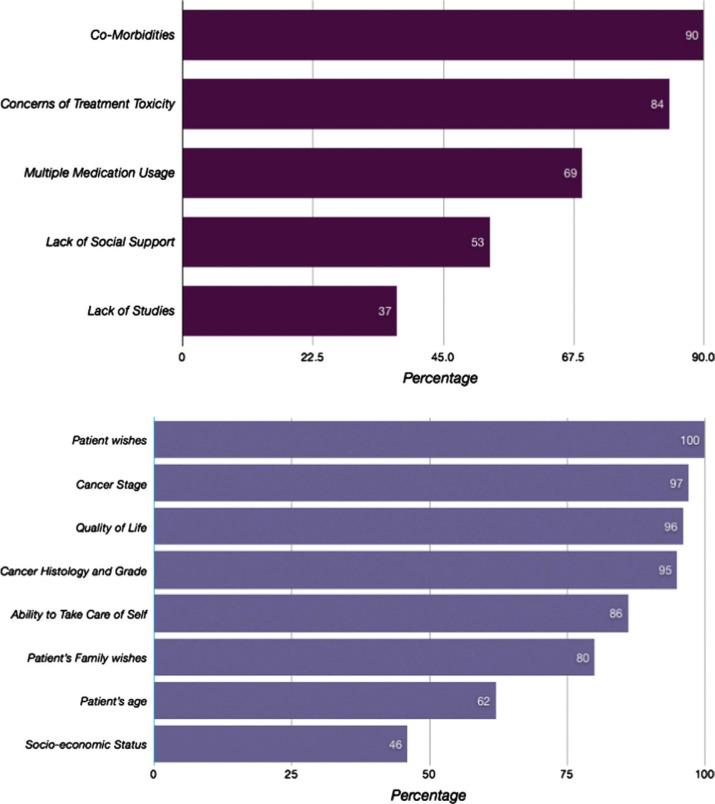
Significant factors and challenges affecting the management of lung cancer in elderly patients: results from a physician provider survey.

Following our review of the above findings, a voluntary confidential survey questionnaire on the physician approach to elderly patient with lung cancer was developed and distributed to our medical staff directly involved in their care. The questionnaire was returned by 34 of 103 primary care providers, 4 of 11 geriatricians, and 27 of 45 internal medicine resident trainees. Responses were similar among the provider groups and are summarized in Fig. 2. Among factors significantly affecting management, 100% of responders respected patient wishes and autonomy; 96% stressed patient quality of life; and 96% noted the importance of cancer histology, grade, and stage. In contrast, 62% of providers reported patient age per se as a concern and 46% reported socioeconomic status as a factor. Among known challenges to providing care to elderly lung cancer patients, 90% felt medical comorbidities were challenging, 84% were concerned about treatment toxicity risks, and 50% noted lack of patient social support as an issue.

## Discussion

Even in the setting of our modern medical technologies, clinical decision-making for the very elderly patients with lung cancer was practiced at our community hospital in lieu of universal invasive diagnostic procedures. The patient–family–physician relationships were key in determining patient management during the first steps of their diagnostic evaluations. For most patients, the lung cancer was unfortunately a short-term end-of-life illness, although approximately 20% of the very elderly have lived with their lung cancer at least 20 months in this series. Cancer registries contain data on the proportions of clinically and pathologically defined lung cancer, yet the relatively large proportion of very elderly patients with simply a clinical diagnosis of lung cancer has not been reported previously in the medical literature to our knowledge. Also, patient level data on diagnostic decision-making has not been reviewed before in the community hospital setting.

Care of our very elderly patients with lung cancer routinely involved the services of our community hospital: physician and nursing care for active symptoms, diagnostic radiology to identify lung lesions, and invasive biopsy techniques for those desiring pathologic tissue diagnosis. Often, the hospital bedside was the site for the first and most important discussion by physician, patient, and family members regarding diagnostic and management decisions. Moreover, this discussion could serve as the first opportunity for our medical trainees to observe and potentially participate in oncologic decision-making in a true life setting. Our hospital medical records defined the primary decision-makers for or against tissue diagnosis and, in addition, often described conversations among those involved. Indeed, as medical providers, we respected patient autonomy and sought out patients’ values, preferences, and goals in these discussions. In addition, the physician's expertise in these discussions on the technical aspects of patient care, risk/benefit analysis, and experience with lung cancer as an illness need not be underplayed. In a recent commentary, Billings and Krakauer have emphasized the dual roles of patient autonomy and physician responsibility as applied to end-of-life care (11). The same balance of input sees well fit for the care of the very elderly with lung cancer.

Are patients, families, and physicians making good diagnostic decisions? Survival here was similar with and without tissue diagnosis. Retrospectively, patients with surgical resection of their lung cancer did live longer as a group although these patients were early stage, highly selected cases, not advanced symptomatic disease as present in the patients in this series without tissue diagnosis.

What should be provided to the very elderly to enable them to cope with their illness with or without direct cancer treatments? Given the combined challenges of lung cancer illness per se, difficult clinical management decisions in the elderly and frequent comorbidities in this patient population, management of the elderly lung cancer patients is best served by input from a multidisciplinary team (8, 12, 13). Primary care physicians, cancer treatment specialists, palliative care specialists, and social workers all can contribute to patient care, sometimes utilizing a patient navigator to guide the patient through the complex medical system (8). Temel et al. (14) have recently shown that early palliative care for older patients with metastatic non-small-cell lung cancer led to improved quality of life and survival.

Are lung cancer patients well cared for in community settings? Surgery, chemotherapy, and radiotherapy are available as well as clinical trials through cooperative research groups. Moreover, the recent development of patient navigators and palliative care services enhance their care.
